# Comparative study of some analytical methods to quantify lignin concentration in tropical grasses

**DOI:** 10.5713/ajas.17.0450

**Published:** 2018-03-02

**Authors:** Alejandro V. Velásquez, Cristian M. M. R. Martins, Pedro Pacheco, Romualdo S. Fukushima

**Affiliations:** 1 Departamento de Nutrição e Produção Animal, Faculdade de Medicina Veterinária e Zootecnia, University of São Paulo, Av. Duque de Caxias, 225 – Pirassununga, SP 13630-900, Brazil

**Keywords:** Lignin, Acetyl Bromide, Degradability, Acid Detergent

## Abstract

**Objective:**

Lignin plays a relevant role in the inhibition of cell wall (CW) structural carbohydrate degradation. Thus, obtaining accurate estimates of the lignin content in tropical plants is important in order to properly characterize the mechanism of lignin action on CW degradation. Comparing conflicting results between the different methods available for commercial use will bring insight on the subject. This way, providing data to better understand the relationship between lignin concentration and implications with tropical forage degradation.

**Methods:**

Five grass species, *Brachiaria brizantha* cv Marandú, *Brachiaria brizantha* cv Xaraés (MG-5), *Panicum maximum* cv Mombaça, *Pennisetum purpureum* cv Cameroon, and *Pennisetum purpureum* cv Napier, were harvested at five maturity stages. Acid detergent lignin (ADL), Klason lignin (KL), acetyl bromide lignin (ABL), and permanganate lignin (PerL) were measured on all species. Lignin concentration was correlated with *in vitro* degradability.

**Results:**

Highly significant effects for maturity, lignin method and their interaction on lignin content were observed. The ADL, KL and ABL methods had similar negative correlations with degradability. The PerL method failed to reliably estimate the degradability of tropical grasses, possibly due to interference of other substances potentially soluble in the KMnO_4_ solution.

**Conclusion:**

ADL and KL methods use strong acid (H_2_SO_4_) and require determination of ash and N content in the lignin residues, therefore, increasing time and cost of analysis. The ABL method has no need for such corrections and is a fast and a convenient method for determination of total lignin content in plants, thus, it may be a good option for routine laboratory analysis.

## INTRODUCTION

Ruminants are highly dependent on forages as a source of energy and a large portion of this energy comes from the plant cell wall (CW) [1]. However, utilization of this energy source may be limited if a substantial portion of what is ingested is not digested, ultimately being excreted in the feces. Lignin has been considered one of the most limiting factors of CW degradation [2], thus, the determination of lignin quantity is essential, to better asses the mechanisms by which lignin inhibits CW degradation.

The more commonly used methods can be classified into two categories: i) those that remove the CW constituents except lignin, and ii) those that breakdown the lignin polymer separating it from the CW structure. In the first category, two procedures that use concentrate (72%) sulfuric acid result in an insoluble acid residue, after hydrolysis of CW polysaccharides. Such is the case of acid detergent lignin (ADL) and Klason lignin (KL) [3]. However, ADL may underestimate lignin due to its partial solubilization in the acid detergent solution (ADS) [4–7] or in the 72% sulfuric acid solution [8]. Also, lignin from the KL method can be contaminated with N residues [8].

In the second category, lignin can be determined by the difference in weight after lignin degradation with oxidizing reagents, such as potassium permanganate. However, this procedure can also oxidize other substances (e.g. pectin, flavonoids, tannins, etc.) that are not completely removed during the fibrous fraction preparation, and are measured as being lignin [9].

An alternative method for lignin quantification that does not fit in neither of the previous categories is the acetyl bromide lignin (ABL) method. In this procedure, solubilized lignin in 25% acetyl bromide solution is read on a spectrophotometer at UV wavelength region of 280 nm [10].

In spite of the various available methods, none of them can be considered as a standard for all types of materials. Knowledge of lignin concentrations help to better understand the degradability of forages, cellulose pulp production and second generation ethanol production. Then, the question of which is the best method for measuring lignin arises. This determination has to be made on the basis of method accuracy and precision, its simplicity, time for analysis and cost per sample, among other factors. The objective of this study was to evaluate ADL, potassium permanganate lignin (PerL), and KL methods for determining lignin in forages and to compare their results to those obtained by the ABL procedure. Also, to correlate results of lignin concentration with *in vitro* degradability.

## MATERIALS AND METHODS

### Samples

Five grass species, *Brachiaria brizantha* cv Marandú, *Brachiaria brizantha* cv Xaraés (MG-5), *Panicum maximum* cv Mombaça, *Pennisetum purpureum* cv Cameroon and *Pennisetum purpureum* cv Napier, were harvested at five maturity stages with 15 day intervals between each cut, after an initial uniformization cut. The maturity stages were pre-flower head emergence 1 (35 days), pre-flower head emergence 2 (50 days), initial flowering (65 days), total flowering (80 days), and post-flowering (95 days).

### Chemical analyses

Fresh samples were weighed and dried in a forced ventilation oven at 60°C, for 72 hours. Dried samples were ground using a Wiley type laboratory knife mill with a 0.5 mm screen. After processing, samples were stored in closed glass containers.

Neutral detergent fiber (NDF) and acid detergent fiber (ADF) (sequentially after NDF) were determined as described by Van Soest et al [11]. To obtain fiber preparations, non-woven textile (NWT; 100 g/m^2^) bags were used. Heat-stable bacterial alpha amylase (Termamyl 2x, activity = 17,400 Liquefon Units/mL, TECNOGLOBO, Curitiba, PR, Brazil) was used to prepare NDF but no sodium sulfite was added. The ADL was determined as described by Van Soest and Robertson [12].

The CW which is used as a fibrous substrate for the ABL procedure was prepared according to the protocol proposed by Fukushima et al [7]. The NDF fraction was corrected for residual ash [13], as well as CW, and crude protein was determined by using the Kjeldahl procedure. Equivalent protein was determined by using the 6.25 factor to convert N content into crude protein.

ADF was used as a fibrous substrate for ADL and PerL analyses. Approximately 500 mg of ADF dry matter (DM) sample was weighed in NWT bags. After ADF extraction, the bags were placed in a beaker and covered with 72% sulfuric acid solution (approximately 30 mL of acid per bag). An Erlenmeyer containing 150 mL of water was placed inside the beaker in order to maintain the bags submerged in the acid solution. The procedure lasted three hours and the acid solution was changed once. Following the acid treatment, bags were carefully rinsed off with distilled water, transferred to another glass beaker containing hot distilled water and left to soak for approximately 12 hours. During this period, water was replenished in order to keep it hot and pH was measured using pH strips to monitor the presence of any acid residue in the bags. Finally, the bags were placed to dry in a forced ventilation oven at 105°C, overnight. After being weighed, bags were incinerated in a muffle furnace in order to correct ADL values for ash presence.

KL was performed as proposed by Hatfield et al [8]. This method is also based on 72% sulfuric acid hydrolysis of CW components, except lignin. Differently from the ADL method, KL uses dietary fiber (DF) as a substrate, instead of ADF. Briefly, approximately 250 mg of DF samples were placed inside 120-mL screw capped bottles. Three milliliters of 12 M sulfuric acid solution were added to each bottle and the contents stirred with a glass rod. The bottles were kept in a 30°C water bath for 60 minutes, after which 80 mL of distilled water was added to each one. Subsequently, the bottles were autoclaved at 105°C for 1 hour. Once autoclaved, the insoluble material was vacuum-filtered in glass crucibles, washed with hot distilled water and dried overnight at 105°C. Finally, ash content was determined.

PerL analysis was performed as described by Van Soest and Wine [9]. Approximately 500 mg of ADF preparation was placed inside glass-crucibles. Each crucible was filled with saturated KMnO_4_ solution (approximately 30 mL/crucible). The content inside the crucible was stirred occasionally using individual glass rods. After filtering under vacuum, this step was repeated once more. The crucibles were filtered and 30 mL of demineralizing solution was added. After 20 minutes, the demineralizing solution was replenished and the crucibles were left to sit for 20 more minutes. Then, the residue was vacuum-filtered and washed three times with approximately 20 mL (each time) of ethanol solution (800 mL/L) and acetone.

The ABL procedure uses CW as a substrate [7]. Approximately 100 mg CW sample were weighed in glass centrifuge tubes fitted with teflon lined caps. A blank control tube was also conducted. Ten mL of a solution of acetyl bromide in acetic acid (250 mL/L) were added. The tubes were then kept in a 50°C water bath for 2 hours, having their contents gently stirred every 30 minutes. After cooling down, the tubes were centrifuged at 2,000×g for 15 minutes. Aliquots (0.5 mL) of the solution were pipetted into test-tubes that contained 6.5 mL of glacial acetic acid and 2 mL of a 0.3 M NaOH solution. After stirring, one milliliter of 0.5 M hydroxylamine hydrochloride solution was added and the contents again stirred.

The absorbance of the solution was determined on a Libra S80 spectrophotometer (BIOCHROM, Cambridge, UK) at a wavelength of 280 nm and inserted in the equation proposed by Fukushima and Kerley [14]:

$$L=\frac{A-0.0009}{23.077}$$

Where *L* is the lignin concentration (mg/mL), *A* is the absorbance (optical density reading) of the unknown sample, 0.0009 is the mean intercept value and 23.077 is the mean extinction coefficient. The resulting *L* value is then multiplied by the CW content of the plant (on a DM basis) and divided by the actual amount of CW utilized after all dilutions (mg of CW weighed divided by 200). This gives the lignin concentration in the plant (g/kg DM).

In the ABL procedure it is recommended that all analysis steps be made in a ventilated hood because the acetyl bromide fumes are irritating for the respiratory tract and vision mucosa. When dosing acetyl bromide solution, it is also recommended to use syringe-type pipettes with positive air displacement, so that the fumes will not damage the pipette. In spite of this, the acetyl bromide reagent is far less corrosive than the 72% H_2_SO_4_ solution used in the other procedures.

### *In vitro* degradation trials

For the degradability trials an adapted Tilley and Terry [15] method was used. Briefly, approximately 500 mg of sample were placed in F-57 bags, 25 μm pore size (ANKOM Technology Corp, Macedon, NY, USA) and incubated in McDougall’s buffer solution and ruminal fluid, at a 4:1 ratio, for 48 hours. After this, a pepsin and hydrochloric acid solution were added and the samples incubated for another 48 hours. The anaerobic environment in the McDougall solution was attained faster by using the technique proposed by Fukushima et al [16].

### Experimental design

A completely randomized experimental design with duplicate analysis for the lignin assays was used. The lignin contents obtained by the different methods were compared between species and maturity stages according to the model:

$$Y_{ijk}=\mu +A_{i}+Sj+M_{k}+AM_{ik}+{ɛ}_{ijk}$$

Where *Y_ijk_* is the dependant variable; *μ* is the general constant; *A_i_* is the effect of the analytical method *i* (random effect); *S_j_* is the effect of the species *j* (fixed effect); *M_k_* is the effect of maturity level *k* (fixed effect); *AM_ik_* is the interaction effect of the analytical method *i* and the maturity level *k*; *ɛ_ijk_* is the random error.

A randomized block design was used for the *in vitro* experiment, with rumen fluid blocked by week. Individual treatment means were compared by Tukey’s test (p<0.05). Correlation coefficients between lignin methods and *in vitro* degradability values were obtained using PROC CORR from SAS 9.1 [17].

The relationship between the lignin concentrations obtained by four different methods and the characteristics of *in vitro* degradability (DM and NDF) were evaluated by linear regression according to the basic model:

$$Y_{ij}=\beta_{0}+\beta_{1}\times S+\beta_{2}\times L_{ij}+\beta_{3}\times (S\times L_{ij})+{ɛ}_{ij}$$

Where *Y_ij_* is the dependent variable observed in maturity level *j* of analytical method *i*; *β_0_* is the intercept; *β_1, 2, 3_* are coefficients for species, lignin and their interaction, respectively; *L_ij_* is the lignin content (g/kg DM); *S* is the variable corresponding to the specie; and *ɛ_ij_* is the random error.

All statistical procedures were performed using PROC MIXED of SAS 9.1 [17] (α = 0.05).

## RESULTS

### Chemical composition

Chemical composition of the five grass species is depicted in Table 1. The CW, NDF, and ADF increased as the plants matured (p<0.0001). For all species at any given maturity, CW values were numerically higher (no statistics run) than the corresponding NDF values. What differentiates CW from NDF is that the latter has the neutral detergent soluble carbohydrates (pectin, β-glucans, galactans, gums, etc.) removed. Then, an estimate of the soluble fiber (SF) fraction is practical (SF = CW − NDF) and aids to better characterize the animal feeds [18]. Estimates of SF concentrations were similar among grass species except for Marandú, which SF concentration was higher (p<0.0001) with mean value of 99.8 g/kg DM.

### Lignin concentrations

The effect for method of lignin quantification was highly significant (p<0.0001) for all plant species as they matured. The interaction between the two variables was significant for Marandú, Xaraés, Cameroon (p<0.0001) and for Napier (p = 0.0003). The interaction was not significant for Mombaça (p = 0.08).

Mean lignin concentrations varied widely among analytical methods. The ADL method yielded the lowest lignin concentrations (p<0.0001) of the four methods. This result was consistently observed in all species at the five maturity stages. Values ranged from 28.1 g/kg DM for *Panicum maximum* cv Mombaça, to 103.1 g/kg DM for *Brachiaria brizantha* cv Xaraés (Table 2).

Lignin values yielded by PerL method ranged from 65.5 g/kg DM to 159.0 g/kg DM for *Brachiaria brizantha* cv Marandú and *Pennisetum purpureum* cv Cameroon, respectively. In this study, KL values ranged from 61.4 g/kg DM to 127.1 g/kg DM for *Brachiaria brizantha* cv Xaraés and *Pennisetum purpureum* cv Napier, respectively. The PerL and KL values observed in this study were approximately double the values observed for ADL. The values for ABL method were the highest among the methods. Values ranged from 89.8 g/kg DM for *Panicum maximum* cv Mombaça to 187.4 g/kg DM for *Brachiaria brizantha* cv Xaraés.

### *In vitro* forage degradability

No differences were observed for IVDMd (p = 0.34) or IVNDFd (p = 0.38) among the grass species at a given maturity. As expected, *in vitro* dry matter degradability (IVDMd) inversely followed maturity stages. Mean values ranged from 487.6 g/kg DM for *Brachiaria brizantha* cv Xaraés to 547.4 g/kg DM for *Pennisetum purpureum* cv Cameroon. *In vitro* neutral detergent fiber degradability (IVNDFd) had the same pattern of IVDMd as the plants matured. Mean IVNDFd values were numerically lower than IVDMd values, ranging from 360.7 g/kg NDF to 430.7 g/kg NDF for *Brachiaria brizantha* cv Marandú and *Pennisetum purpureum* cv Napier, respectively.

### Correlations

Negative correlations between lignin contents and IVDMd or IVNDFd were observed for all four methods. Higher correlations were observed for IVDMd than for IVNDFd. Correlations for IVDMd ranged from −0.69 (*r*<0.0001) for PerL to −0.81 (*r*<0.0001) for KL. For IVNDFd, correlations ranged from −0.54 (*r*<0.0001) to −0.68 (*r*<0.0001) for PerL and KL, respectively.

## DISCUSSION

### Chemical composition

Increase in the DM content is due, primarily, to increase of CW structural elements [1]. As plants mature, the CW fraction gradually increases and so does the DM content, considering that up to 80% of all DM is composed of CW. This was observed by the increased values of fibrous fractions (NDF, ADF, and CW) (Table 1).

Ash and protein contents were measured in CW and NDF fractions. These substances have long been known to be present in these fibrous fractions and the corrections are made simply because they are not considered fiber, from a nutritional point of view, even though protein plays a key structural role inside the plant cellular wall [19]. Values of ash and protein residues in the CW and NDF fractions were low, as was expected for grass species. Obviously, for both fibrous fractions, the corrected values were lower than their corresponding uncorrected values. Although the values for ash/protein residues were not high, in some cases like Marandú and Cameroon they represented up to 10% of the CW fraction (Obs: these grasses were cultivated in experimental plots, thus soil contamination may not have played an important role).

Differences between CW and NDF were on account of SF loss during NDF extraction. The CW preparation preserves the majority of its components [20], because only water and organic solvents are used to extract the cell solubles. On the other hand, NDF preparation uses a detergent that solubilizes pectin and other polysaccharides (such as β-glucans, galactans, gums, etc.). These neutral detergent soluble carbohydrates are referred to as SF [21]. Given its importance, Queiroz et al [18] proposed the creation of a specific carbohydrate fraction, (B_2_), for SF if using the Cornell Net Carbohydrate and Protein System. Generally speaking, SF is a minor component of the CW structure; however, for some species, like *Brachiaria brizantha* cv Marandú, this fraction represented as much as 20% of the total CW components (Table 1). This aspect may be advantageous to the animal for pectin is almost completely degraded in the rumen [11].

### Lignin concentrations

The gravimetric method ADL is probably the most widely used method for lignin determination in Agronomy and Animal Sciences. Our study showed low lignin values (Table 2). Several others [2,3,8,22] have consistently reported that the values obtained by this method are lower than values obtained by other procedures.

These lower values may be due to partial solubilization of lignin in ADS [4,5,7,23]. Lignin is located inside the CW, covalently linked to hemicellulose, surrounded by cellulose in a tridimensional structure [24]. The dissolution of hemicellulose by the ADS leaves lignin in a loose arrangement from which part of it can be removed and later lost during washing [6].

On the other hand, KL has been known to be contaminated with protein or structural carbohydrate residues that are not completely hydrolyzed [8]. These authors reported that KL values could be as much as three times ADL values because of the combination involving loss of lignin in the ADL method plus protein and carbohydrate contamination in the KL method. The present study showed that KL values were, on average, 1.7 times ADL values. According to Van Soest [1] some tanniferous compounds are solubilized by the ADS, whereas KL can contain a variety of condensed tannins and tannin-protein compounds. In some species, specially legumes, these compounds can contribute to the differences observed between ADL and KL.

Another analytical method for lignin quantification, the PerL procedure uses the same fibrous substrate as the ADL method does. PerL values were higher than ADL concentrations, varying from 1.8 to 2.4 times (Table 2). The PerL values have been known to be affected by pectin presence in the ADF residue [9]. Usually grasses contain relatively low concentrations, so the potential for interference is considered low. However, Table 1 shows the values of SF ranging from 25.3 to 99.8 g/kg of DM. Considering that pectin probably constitutes more than half of the SF fraction it could impact PerL values. In spite of these findings, the PerL method is still used by researchers who prefer avoiding utilization of highly corrosive 72% sulfuric acid.

The spectrophotometric method ABL was initially developed for the wood industry and later modified for its use in forages [10]. Until recently one important setback was the lack of an acceptable standard to which readings of unknown samples could be compared to [20]. Then, Fukushima and Kerley [14] proposed a regression equation, obtained from the calibration curves of several plants, that can be used to determine the lignin content in any plant species. The present study showed the highest lignin values for the ABL method. However, the supposedly high lignin concentrations could be caused by esters of hidroxycinamic acids that are loosely bound to the plant CW [25]. Being a spectroscopic method, ABL would be able to detect soluble lignin components that would be lost in conventional gravimetric methods. The presence of these acids could explain the high absorbance values [26], especially in grass species where they are found in higher concentrations [27].

Morrison and Stewart [26] had mentioned that this difference may be the reason why distinct regression equations had to be used for grasses and legumes to convert absorbance values to lignin contents. However, recent data [7] relating ABL method with *in vitro* degradability assays showed that slopes between grasses and legumes had similar inclinations.

Another question that arises around the ABL method is that it could detect other phenol-containing compounds (protein, tannins, flavonoids) that absorb UV light in the same region where lignin absorbs (280 nm). The ABL method uses the fibrous preparation CW [20]. During its preparation, tannins and flavonoids are removed [10], therefore these compounds would not pose a problem. In regards to a possible interference of CW protein in the absorbance readings, Morrison [10] analyzed the precipitate formed after CW digestion in the 25% acetyl bromide solution and concluded that it was mainly protein. Fukushima found protein content in this precipitate varying from 515.4 to 693.0 g/kg; the remaining sediment was mineral matter (data not published). Thus, these substances probably do not interfere with the optical density readings used to determine lignin concentration.

### Correlation with *in vitro* forage degradability

Correlation between lignin concentration measured by a specific analytical method and an *in vitro* degradability (Table 3) assay is one tool that can assist in the characterization of an analytical method. The chemical accuracy of nutrient determination improves the degree of correlation and is therefore of critical importance [28]. Even though the degree of correlation may be unsatisfactory for some situations, this index reflects the practicality of being able to obtain a more accurate estimate of nutritive value from chemical compositional data [12].

Figure 2 shows that correlations within IVNDFd were lower than the correlations within IVDMd. It was expected that expressing lignin per unit of NDF would improve its correlation with CW degradability, but this study showed the opposite. Because lignin impacts only CW degradation, not non-CW components (non-NDF components are virtually 100% degradable), it has been suggested that expressing lignin on a DM basis should be avoided [1]. Jung and Vogel [29] found that lignin as a percentage of DM gave an equal or better fit to the model than lignin as a percentage of NDF, and attributed their observations to a greater precision in determination of DM compared to NDF. In addition, Traxler et al [30] reported a better correlation between the unavailable residue of NDF and the lignin concentration of DM, rather than the lignin concentration of NDF.

The PerL method gave the lowest correlation coefficients then, it may not reliably estimate the degradability of tropical grasses. This may be caused by the interference of substances such as pectin, tannins or flavonoides that are oxidized by the KMnO_4_ solution [9]. In an experiment conducted in our laboratory (data not published), the tannin and total phenols contents were quantified in crude CW of grasses (9.5 to 35.5 g/kg DM) and legumes (11.3 to 80.6 g/kg). Therefore, they could appear as an artifact of the PerL technique. According to Van Soest and Wine [9], these phenolic compounds could represent a bias mainly in tanniferous plants. Also, PerL uses ADF as substrate for lignin analysis and therefore suffers from the same restrictions as the ADL method that were previously discussed.

**Figure 1:**
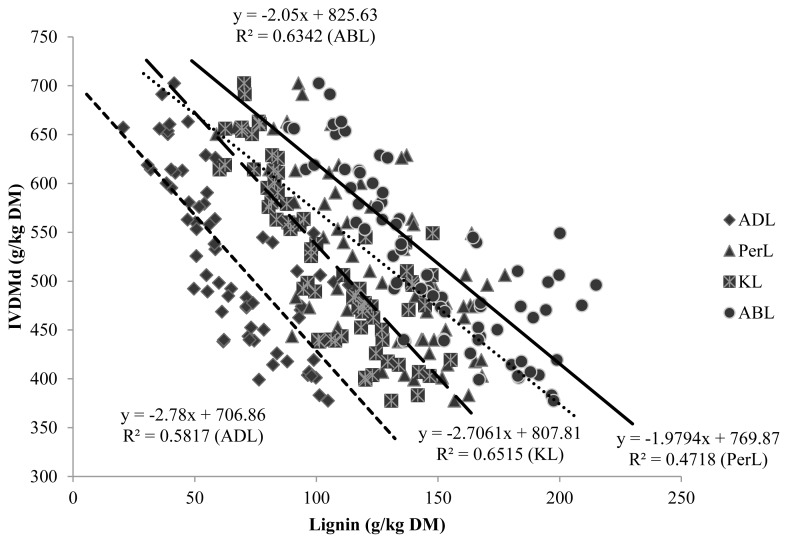
Regression parameters for the relationship between lignin contents, obtained by different methods, and *in-vitro* DM digestibility. DM, dry matter; ADL, acid detergent lignin; PerL, permanganate lignin; KL, Klason lignin; ABL, acetyl bromide lignin.

**Figure 2:**
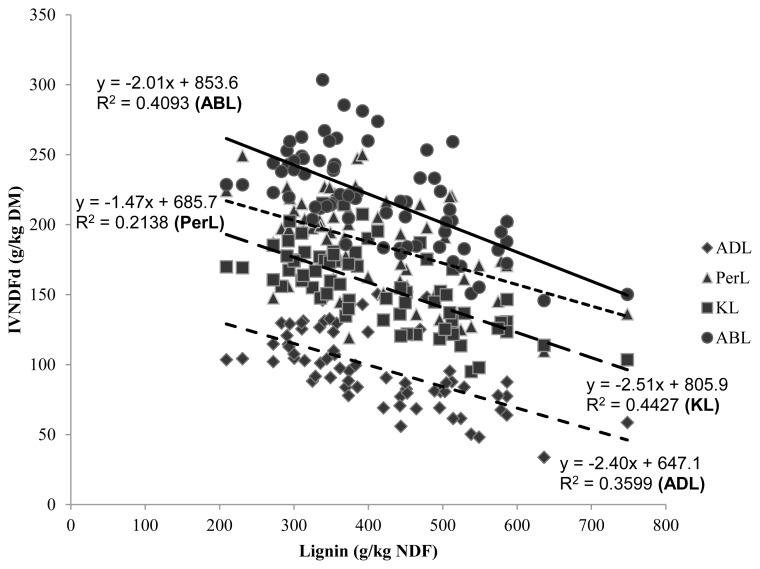
Regression parameters for the relationship between lignin contents, obtained by different methods, and *in-vitro* NDF digestibility. NDF, neutral detergent fiber; ADL, acid detergent lignin; PerL, permanganate lignin; KL, Klason lignin; ABL, acetyl bromide lignin.

Even though ADL and KL methods yielded different lignin values, they had similar correlations with degradability and therefore, could estimate degradability of forages with comparable accuracy. Figures 1 and 2 illustrate that the slopes of both methods were much the same and curves were parallel. Similar correlation coefficients were also obtained by Jung et al [3]. These authors found that both lignin methods were generally negatively correlated with DM and NDF degradability in both *in vitro* and *in vivo* systems. They stated that, both methods had similar degree of correlation with degradability of forages but, KL could produce a more accurate measurement of total lignin content than ADL, especially in grasses. Hatfield et al [8] found conflicting concentrations for the ADL and KL methods but suggested that the KL method could produce more accurate results.

This work showed that the ABL method had comparably good correlations. Fukushima et al [7] reported highest correlation coefficients for ABL when compared to other analytical methods, for both grass and legume degradation.

The pattern observed for *in vitro* forage degradability values confirms the physiological changes in the composition of forages as they mature and the implications of these changes on microbial degradation. Lignin concentration was linearly associated to IVDMd and IVNDFd (Figures 1, 2, respectively). This had already been reported by other authors [13, 24]. The elevated correlations observed in this study inferred that the methods ADL, KL and ABL ranged evenly within the five species and five maturity stages.

## CONCLUSION

Even though this study showed varying results obtained by different lignin methods, it is important to notice that correlations were high between degradability of DM and the methods ADL, KL, and ABL. However, both ADL and KL methods use strong acids that require adequate manipulation and it is recommended that ash be determined in the lignin residues, which increases labor and time of analysis. Nitrogen contamination in the KL residue is also advisable to quantify. In the ABL method there is no need for such corrections. Further studies, involving a wider range of species including legumes, are needed. Nevertheless, the ABL method is convenient for determination of total lignin content in plants and a good option for routine laboratory analysis.

## IMPLICATIONS

This study provides data to better understand the relationship between lignin concentration and implications with tropical forage degradation. It is also an effort towards the standardization of a reliable, safe and fast method for lignin quantity assays. The spectrophotometric method is a safer procedure when compared against sulfuric acid lignin methods. Information about its use can stimulate interest in the method by the scientific community and allow for expansion of its use in routine laboratory analysis. An accurate procedure for lignin quantification could help to better understand how ruminant animals use forages and improve milk and meat production.

## Figures and Tables

**Table 1:** Mean chemical composition (g/kg DM) of five grass species at different maturity levels

Maturity	CW1)	NDF1)	SF2)	IVDMd	IVNDFd
	*Brachiaria brizantha* cv Marandú				
1	586.7	481.3	107.6	652.2	487.9
2	645.2	543.9	101.3	612.2	454.0
3	666.6	565.4	101.2	556.8	337.1
4	692.7	595.8	96.9	439.1	220.0
5	720.2	621.9	98.3	478.3	275.0
Mean	662.3	561.7	101.1	547.7	354.8
SE	13.9	14.7	1.6	19.6	24.5
	*Brachiaria brizantha* cv Xaraés				
1	695.2	636.3	58.9	616.6	543.7
2	722.5	688.8	33.9	571.8	480.2
3	747.1	718.9	28.2	509.0	431.5
4	782.2	753.9	28.4	448.1	360.0
5	795.1	769.2	25.9	402.5	303.8
Mean	748.4	713.4	35.1	509.6	423.8
SE	13.9	16.9	3.5	22.2	23.7
	*Panicum maximum* cv Mombaça				
1	642.8	607.0	35.9	656.8	580.4
2	692.0	656.5	35.5	604.9	516.0
3	722.7	689.1	33.5	595.4	509.0
4	745.7	713.1	32.0	493.9	372.2
5	749.4	717.2	32.3	439.7	307.4
Mean	710.5	676.6	33.8	558.1	457.0
SE	13.9	15.0	1.9	26.5	30.6
	Pennisetum purpureum cv Cameroon				
1	596.5	539.9	56.6	697.0	584.0
2	647.3	617.5	29.6	627.7	511.1
3	666.5	644.5	22.0	560.8	436.9
4	670.3	653.0	17.4	499.2	384.1
5	703.0	685.0	17.2	475.8	349.1
Mean	656.7	628.0	28.6	572.1	453.0
SE	12.3	15.4	4.2	22.2	23.1
	*Pennisetum purpureum* cv Napier				
1	671.0	613.7	57.4	662.0	616.0
2	688.5	642.0	46.5	577.6	519.3
3	704.1	667.8	36.2	535.4	455.5
4	726.5	693.8	32.7	476.8	371.6
5	740.4	716.8	24.6	445.2	340.1
Mean	706.1	666.8	39.5	539.4	460.5
SE	10.5	13.9	4.0	22.8	28.8

DM, dry matter; CW, cell wall; NDF, neutral detergent fiber; SF, soluble fiber; IVDMd, *in vitro* dry matter degradability; IVNDFd, *in vitro* neutral detergent fiber degradability; SE, standard error of the mean.

1) Corrected for ash and protein.

2) SF = CW − NDF.

**Table 2:** Lignin concentrations (g/kg DM) and mean SE obtained through four analytical procedures

Maturity	ADL	KL	PerL	ABL
	*Brachiaria brizantha* cv Marandú			
1	39.0^Cc^	71.5^Db^	65.5^Ec^	110.0^Ca^
2	43.5^BCd^	83.4^Cc^	98.3^CDb^	117.8^Ca^
3	50.6^Bc^	89.0^Cb^	110.8^Ba^	118.1^Ca^
4	61.9^Ac^	101.0^Bb^	141.1^Aa^	136.2^Ba^
5	71.3^Ad^	116.2^Ab^	94.4^Dc^	151.4^Aa^
Mean	53.3	92.2	102.0	126.7
SE	0.4	1.0	5.1	0.9
	*Brachiaria brizantha* cv Xaraés			
1	31.3^Dc^	61.4^Db^	94.9^Ba^	97.4^Da^
2	47.4^Cc^	82.6^Cb^	92.3^Bb^	127.0^Ca^
3	50.2^Cd^	96.3Bc	118.8^Cb^	131.8^Ca^
4	72.7^Bd^	114.4^Ac^	99.4^Bb^	167.0^Ba^
5	98.2^Ad^	121.5^Ac^	144.0^Ab^	187.4^Aa^
Mean	60.0	95.2	109.9	142.1
SE	0.7	1.9	7.2	1.4
	*Panicum maximum* cv Mombaça			
1	28.1^Ec^	66.0^Eb^	74.6^Cab^	89.8^Da^
2	40.4^CDc^	77.2^DEb^	83.1^Cb^	112.9^Ca^
3	46.8^BCc^	83.5^CDb^	113.1^Ba^	125.2^BCa^
4	57.6^Bc^	98.0^BCb^	134.7^Aa^	139.2^ABa^
5	73.6^Ad^	107.0^ABc^	139.5^Ab^	152.4^Aa^
Mean	49.3	86.3	109.0	123.9
SE	3.8	1.9	5.1	2.1
	*Pennisetum purpureum* cv Cameroon			
1	39.1^Cc^	70.4^Cb^	93.5^Ca^	103.3^Ea^
2	56.5^Bc^	83.0^Bb^	136.1^Ba^	127.7^Da^
3	57.2^Bc^	93.9^Bb^	139.7^Ba^	133.5^CDa^
4	59.9^Bd^	114.5^Ac^	155.1^Aa^	144.2^BCb^
5	72.6^Ac^	119.6^Ab^	159.0^Aa^	167.5^Aa^
Mean	57.1	96.3	136.7	135.2
SE	2.4	1.2	2.9	1.2
	*Pennisetum purpureum* cv Napier			
1	43.5^Cd^	76.6^Dc^	97.1^Db^	108.7^Ea^
2	52.9^BCc^	84.1^Db^	113.0^Ca^	121.2^Da^
3	58.3^Bc^	99.0^Cb^	135.7^Ba^	134.9^Ca^
4	62.5^Bc^	117.5^Bb^	143.1^ABa^	150.4^Ba^
5	75.5^Ad^	127.1^Ac^	148.5^Ab^	170.5^Aa^
Mean	58.5	100.9	127.5	137.1
SE	1.9	1.3	5.2	2.4

DM, dry matter; SE, standard error of the mean; ADL, acid detergent lignin; KL, Klason lignin; PerL, permanganate lignin; ABL, acetyl bromide lignin.

Means by specie, within columns for maturity followed by different capital letters or, within rows for analitycal method followed by different lower-case letters are different according to Fisher’s LSD (p<0.05).

**Table 3:** Correlations between lignin contents (g/kg DM) obtained by different methods and IVDMD and IVNDFD

Method	Method					

	ADL	KL	PerL	ABL	IVDMD	IVNDFD
**ADL**	1.0	-	-	-	-	-
**KL**	0.9352	1.0	-	-	-	-
*r*	<0.0001	-				
**PerL**	0.7324	0.7425	1.0	-	-	-
*r*	<0.0001	<0.0001	-	-		
**ABL**	0.9750	0.9594	0.7498	1.0	-	-
*r*	<0.0001	<0.0001	<0.0001	-	-	-
**IVDMD**	−0.7627	−0.8072	−0.6869	−0.7964	1.0	-
*r*	<0.0001	<0.0001	<0.0001	<0.0001	-	-
**IVNDFD**	−0.6140	−0.6822	−0.5356	−0.6367	0.8953	1.0
*r*	<0.0001	<0.0001	<0.0001	<0.0001	<0.0001	-

DM, dry matter; IVDMD, *in vitro* dry matter degradability; IVNDFD, *in vitro* neutral detergent fiber degradability; ADL, acid detergent lignin; KL, Klason lignin; PerL, permanganate lignin; ABL, acetyl bromide lignin.
